# circUBE2G1 interacts with hnRNPU to promote VEGF-C-mediated lymph node metastasis of lung adenocarcinoma

**DOI:** 10.3389/fonc.2024.1455909

**Published:** 2024-11-27

**Authors:** Yuting Li, Hui Chen, Yue Zhao, Qilu Yan, Lulu Chen, Qibin Song

**Affiliations:** ^1^ Cancer Center, Renmin Hospital of Wuhan University, Wuhan, Hubei, China; ^2^ Department of Emergency Medicine, Sun Yat-sen Memorial Hospital, Sun Yat-sen University, Guangzhou, Guangdong, China; ^3^ Guangdong Provincial Key Laboratory of Malignant Tumor Epigenetics and Gene Regulation, Sun Yat-sen Memorial Hospital, State Key Laboratory of Oncology in South China, Guangzhou, Guangdong, China; ^4^ Department of Interventional Oncology, The First Affiliated Hospital of Sun Yat-sen University, Guangzhou, Guangdong, China

**Keywords:** lung adenocarcinoma, vascular endothelial growth factor C, lymphangiogenesis, lymph node metastasis, circular RNAs

## Abstract

**Background:**

Patients with lymph node(LN)metastasis-positive Lung adenocarcinoma(LUAD)suffer from a significantly reduced five-year survival rate. Increasing evidence indicates circular RNAs(circRNAs)play crucial roles in regulating cancer progression. However, the specific regulatory mechanisms of circRNAs in the LN metastasis of LUAD have not been fully explored.

**Methods:**

GEO datasets and sequence analysis were applied for the identification of differentially expressed circRNAs between LUAD tissues and adjacent normal tissues. *In vitro* and *in vivo* experiments were performed to evaluate the function of circUBE2G1. The interaction between circUBE2G1 and VEGF-C was determined by RNA pulldown, ChIP, ChIRP and luciferase assays.

**Results:**

In this study, we identified a novel circRNA, circUBE2G1 (hsa_circ_0041555), which is upregulated in LUAD and positively correlated with LN metastasis in patients with LUAD. Functionally, overexpression of circUBE2G1 promotes lymphangiogenesis and LN metastasis of LUAD both *in vitro* and *in vivo*. Mechanistically, circUBE2G1 activates the transcription of vascular endothelial growth factor C (VEGF-C) by recruiting hnRNPU to enhance H3K27ac on the VEGF-C promoter, thereby facilitating lymphangiogenesis and LN metastasis in LUAD.

**Conclusion:**

Our findings offer new insights into the mechanisms behind circRNA-mediated LN metastasis in LUAD and suggest that circUBE2G1 may serve as a potential therapeutic target for LN metastasis in LUAD.

## 1 Introduction

Lung cancer is the second most prevalent type of cancer and has become a leading cause of cancer-related deaths globally. Despite improvements in therapeutic approaches, the prognosis of lung cancer remains poor with an average five-year survival rate of merely 22% (1). Approximately 40% of lung cancers are lung adenocarcinomas (LUAD), making it the most common histological type (2). Lymph node (LN) status is a primary determinant of staging and prognosis in patients with LUAD. Significant differences in five-year survival rates have been observed based on the extent of LN metastasis (3). Despite the clinical significance of LN metastasis in LUAD, the underlying mechanisms remain unclear.

Circular RNAs (circRNAs) are a class of small non-coding RNAs characterized by a covalently closed loop structure (4). CircRNAs are widely expressed in various cancers and play crucial roles in regulating cancer progression (5). They act as transcriptional regulators, microRNA sponges, and protein translation templates (6–8). Kong et al. found that circNFIB1 inhibits lymphangiogenesis and LN metastasis in pancreatic cancer by targeting the miR-486-5p/PIK3R1/VEGF-C axis (9). Li et al. discovered that the peptide translated from circFBXW7 inhibits the classical Wnt signaling pathway, affecting the sensitivity of LUAD to tyrosine kinase inhibitors (TKIs) (10). However, the specific regulatory mechanisms of circRNAs in the LN metastasis of LUAD have not been fully explored.

Lymphangiogenesis is defined as the formation of new lymphatic vessels from pre-existing ones. It is a crucial rate-limiting step in the LN metastasis of tumor cells (11). Accumulating evidence suggests that tumors actively induce lymphangiogenesis, and the number of lymphatic vessels is closely associated with the clinical outcomes of various types of cancer (12). Several factors were shown to promote lymphangiogenesis in cancer, among which vascular endothelial growth factor C (VEGF-C) is the most prominent and extensively studied lymphangiogenic factor (13). VEGF-C promotes the migration, proliferation, and survival of lymphatic endothelial cells by binding to vascular endothelial growth factor receptor 3 (VEGFR3) (14, 15). Despite the crucial role of VEGF-C in lymphangiogenesis and LN metastasis, little is known about how VEGF-C expression is regulated and how VEGF-C induces lymphangiogenesis in LUAD.

This study found that circUBE2G1 (hsa_circ_0041555) is significantly upregulated in LUAD, which is positively correlated with LN metastasis in patients with LUAD. Functionally, it was found that circUBE2G1 promotes lymphangiogenesis and LN metastasis in LUAD. Mechanistically, circUBE2G1 activates VEGF-C transcription by interacting with hnRNPU, thereby facilitating lymphangiogenesis and LN metastasis in LUAD. These findings reveal that circUBE2G1 may serve as a therapeutic target for LN metastasis in LUAD.

## 2 Results

### 2.1 circUBE2G1 is correlated with LN metastasis in LUAD

**Figure 1:**
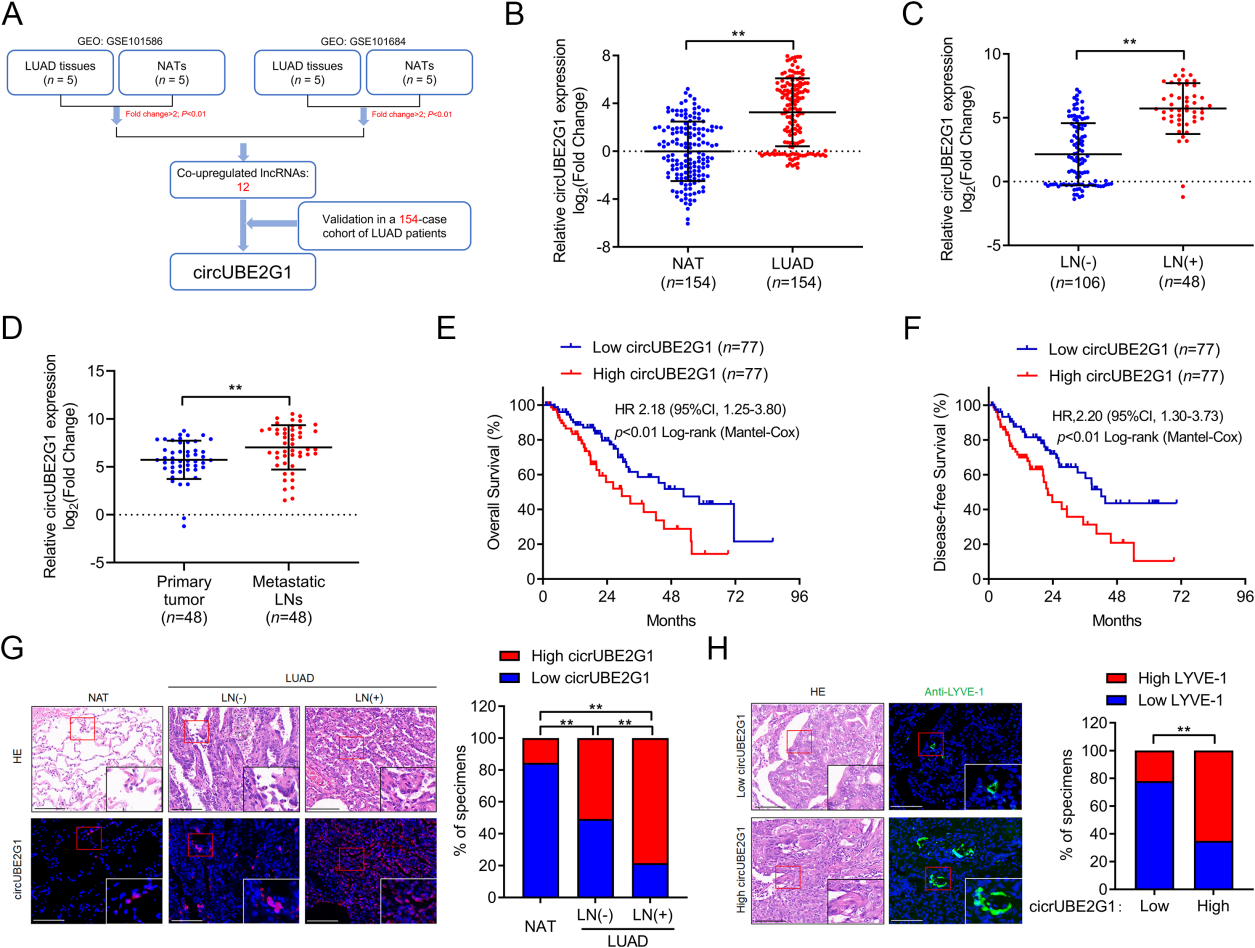
CircUBE2G1 overexpression is positively correlated with LN metastasis in LUAD. **(A)** Schematic diagram of the circRNAs screening process. **(B)** qRT-PCR analysis of circUBE2G1 expression in LUAD and NATs. **(C)** qRT-PCR analysis of circUBE2G1 expression in LUAD with negative or positive lymph node metastasis. **(D)** qRT-PCR analysis of circUBE2G1 expression in primary LUAD tissues and paired metastatic lymph nodes. **(E, F)** Kaplan-Meier curves of OS **(E)** or DFS **(F)** in patients with LUAD based on circUBE2G1 expression. The median expression was chosen as the cutoff value. **(G)** FISH images and percentages of circUBE2G1 expression in NAT, LN-negative LUAD, and LN-positive LUAD tissues. Scale bars, 50 μm. **(H)** Representative images and percentages of LYVE-1-positive lymphatic vessels in LUAD tissues with low and high expression of circUBE2G1. Scale bars, 50 μm. Statistical differences were assessed using the non-parametric Mann-Whitney U test in **(B–D)** and using the Chi-square test in **(G, H)**. Error bars represent the standard deviation of three independent experiments. **P < 0.01.

LUAD tumor tissues and paired adjacent non-tumor tissues (NATs) from the public GEO datasets (GSE101586 and GSE101684) were analyzed to identify key circRNAs promoting LN metastasis in LUAD. The intersection of these two sequencing experiments indicated 12 circRNAs significantly upregulated in LUAD tissues (**Figure 1A**). Subsequent analysis of a cohort of 154 patients with LUAD revealed that circUBE2G1 (hsa_circ_0041555) was markedly overexpressed in LUAD tissues compared to NATs (**Figure 1B**). Compared to LN-negative LUAD tissues (*n*=106), circUBE2G1 was overexpressed in LN-positive samples (*n*=48) (**Figure 1C**). Metastatic LNs had higher expression levels of circUBE2G1 compared to primary tumors (**Figure 1D**). Using the median as a cutoff value, Kaplan-Meier analysis demonstrated that high circUBE2G1 expression was associated with poorer overall survival (OS) and disease-free survival (DFS) in patients with LUAD (**Figures 1E, F**). Fluorescence *in situ* hybridization (FISH) of LUAD tissues also confirmed this differential expression (**Figure 1G**; **Supplementary Figure S1A**). In addition, a high density of lymphatic vessels positive for lymphatic vessel endothelial hyaluronan receptor 1 (LYVE-1) were found in both intratumoral and peritumoral areas of LUAD with high expression of circUBE2G1 (**Figure 1H**). Overall, these results suggest that circUBE2G1 is positively correlated with LN metastasis and poor prognosis in LUAD.

### 2.2 Identification of circUBE2G1 characteristics

**Figure 2:**
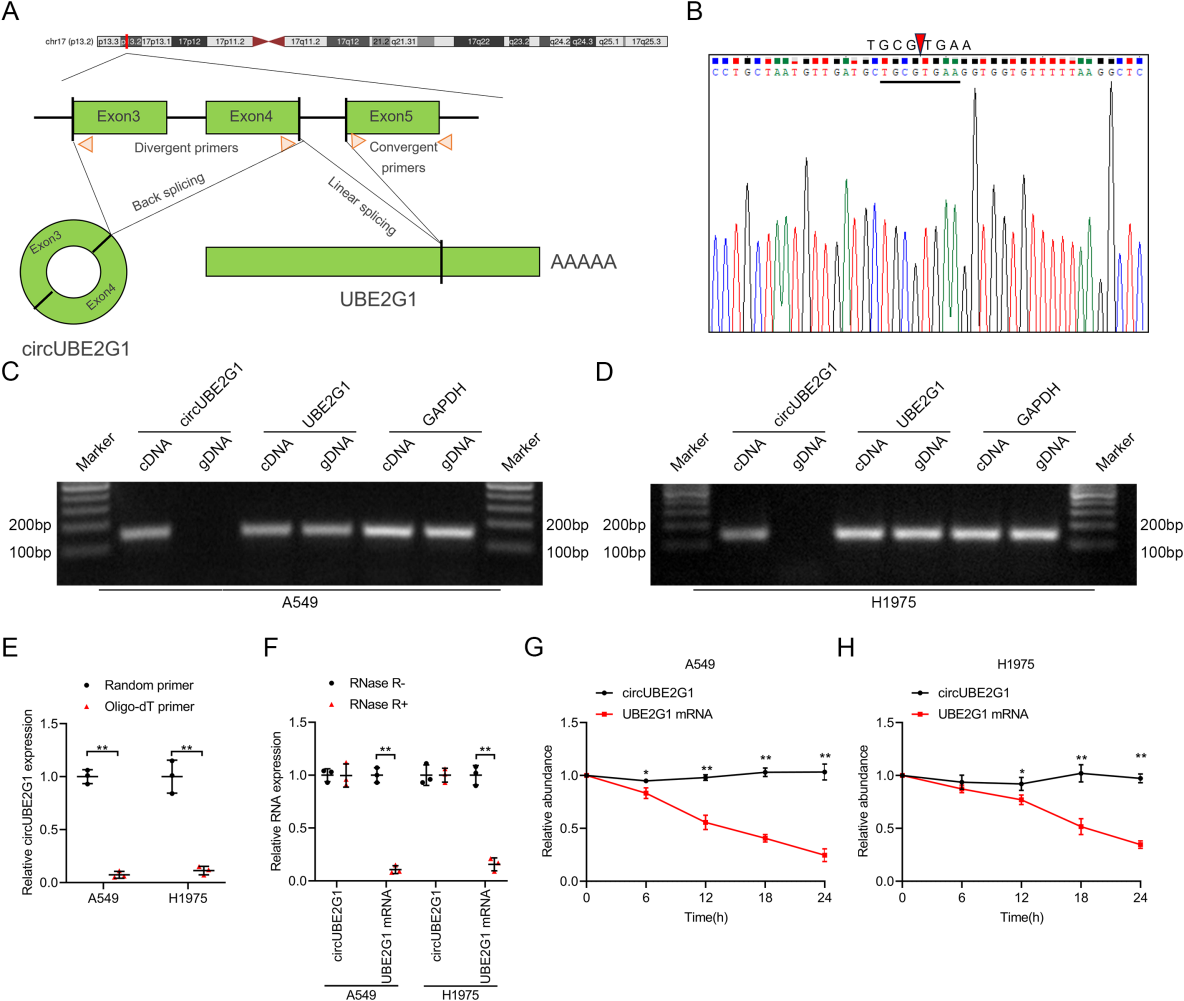
Identification of circUBE2G1 characteristics. **(A)** Schematic diagram of circUBE2G1 splicing. **(B)** Sanger sequencing for circUBE2G1. **(C, D)** PCR and agarose gel electrophoresis measuring circUBE2G1 and UBE2G1 in cDNA and gDNA of A549 cells **(C)** and H1975 cells **(D)**. **(E)** qRT-PCR analysis of circUBE2G1 expression using random primers or oligo-dT primers. **(F)** qRT-PCR analysis of circUBE2G1 and UBE2G1 expression in LUAD cells with or without RNase R treatment. **(G, H)** Evaluation of circUBE2G1 and UBE2G1 mRNA stability in A549 cells **(G)** and H1975 cells **(H)**. Statistical differences were measured using two-tailed student t-test in **(E–H)**. Error bars represent the standard deviation of three independent experiments. *P < 0.05; **P < 0.01.

Sequence analysis and Sanger sequencing of circUBE2G1 revealed that it forms by back-splicing of exons 3 to 4 of the UBE2G1 transcript (**Figures 2A, B**). Using complementary DNA (cDNA) and genomic DNA (gDNA) from LUAD cells as templates, divergent primers amplified circUBE2G1 in the cDNA group, not in the gDNA group (**Figures 2C, D**). Reverse transcription using oligo-dT primers instead of random primers significantly reduced circUBE2G1 expression, indicating the absence of a poly(A) tail (**Figure 2E**). Furthermore, treatment with RNase R to total RNA significantly decreased the mRNA levels of UBE2G1, but not circUBE2G1. These findings confirm that circUBE2G1 is composed of a closed circular structure (**Figure 2F**). Treatment of LUAD cell lines with actinomycin D showed that the half-life of circUBE2G1 was longer than that of UBE2G1 mRNA, confirming its stability (**Figures 2G, H**). Collectively, these results indicate that circUBE2G1 is a highly stable circRNA.

### 2.3 circUBE2G1 promotes the lymphangiogenesis of LUAD *in vitro*

**Figure 3:**
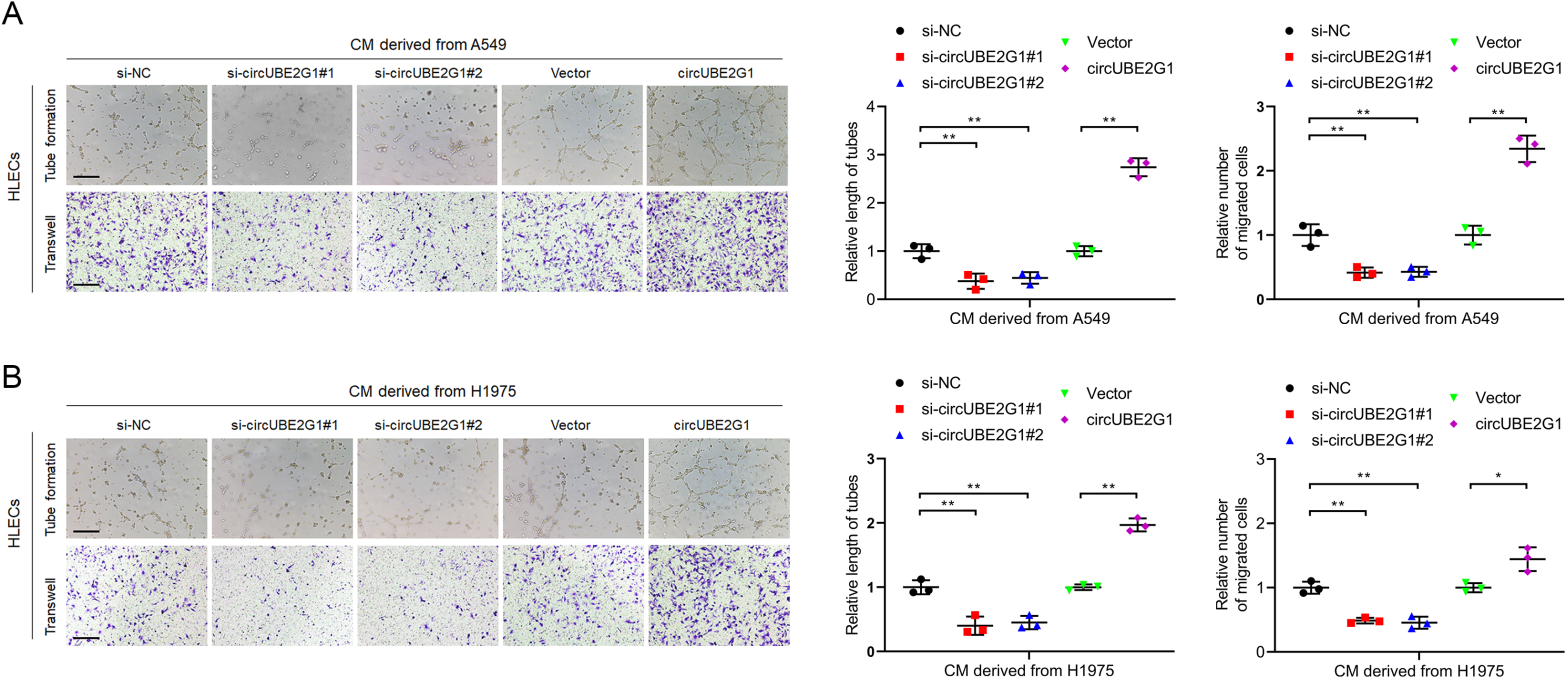
CircUBE2G1 promotes lymphangiogenesis *in vitro*. **(A, B)** Representative images and quantification of tube formation and migration of HLECs treated with culture medium from A549 **(A)** or H1975 **(B)** cells with circUBE2G1 knockdown or overexpression. Scale bars, 100 μm. Statistical significances between two groups were assessed using two-tailed t-test, and statistical significances between multiple groups was assessed using one-way ANOVA followed by Dunnett’s t-test. Error bars represent the standard deviation of three independent experiments. *p < 0.05, **p < 0.01.

Given the clinical association of circUBE2G1 with LN metastasis in LUAD, we investigated the *in vitro* effects of circUBE2G1 on lymphangiogenesis in LUAD. We successfully altered circUBE2G1 expression via transfection with siRNAs or circUBE2G1 plasmid, whereas no obvious change was observed in the UBE2G1 mRNA level (**Supplementary Figures S1B–E**). Subsequent co-culture of human lymphatic endothelial cells (HLECs) with LUAD cells demonstrated that tube formation and migration of HLECs were significantly inhibited in the circUBE2G1 knockdown group compared to the control group. On the contrary, co-culturing with LUAD cells overexpressing circUBE2G1 significantly promoted the tube formation and migration of HLECs (**Figures 3A, B**). Overall, these results reveal that circUBE2G1 inhibits the lymphangiogenesis of LUAD *in vitro*.

### 2.4 circUBE2G1 facilitates the LN metastasis of LUAD *in vivo*

**Figure 4:**
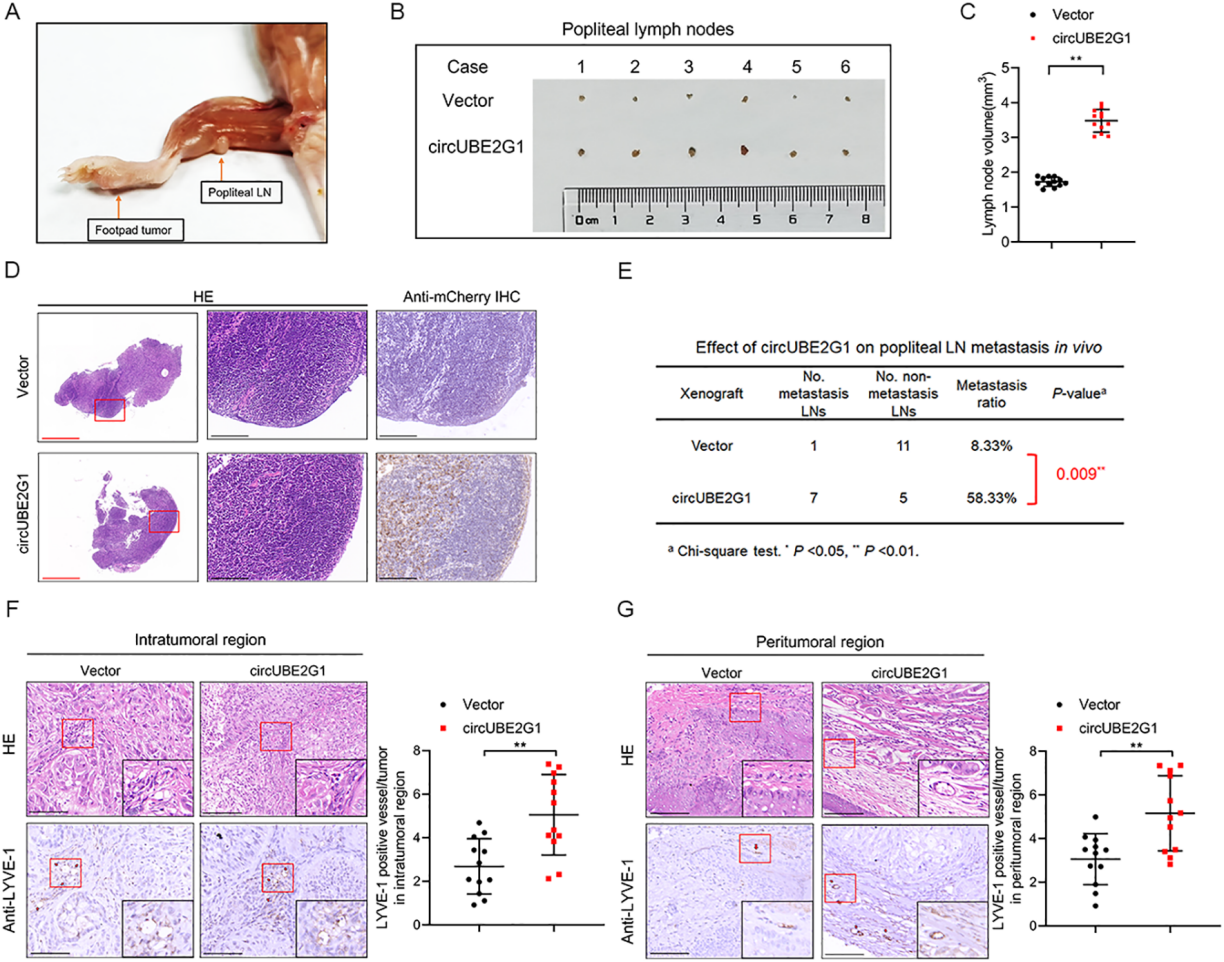
CircUBE2G1 promotes the LN metastasis of LUAD *in vivo*. **(A)** Representative images of popliteal LN metastasis in nude mice. **(B, C)** Representative images **(B)** and histogram **(C)** for popliteal LN volume (*n*=12 per group). **(D)** Representative images of anti-mCherry immunohistochemistry analysis for popliteal LNs (*n* = 12 per group). Red scale bars, 500 μm; black scale bars, 100 μm. **(E)** Popliteal LN metastasis rate in nude mice (*n* = 12 per group). **(F, G)** Representative IHC images showing the percentages of LYVE-1 and lymphatic vessel density in primary tumor tissues. Scale bars, 50 μm. Statistical differences were evaluated using two-tailed t-test **(C, F, G)** and χ2 test **(E)**. Error bars represent the standard deviation for three independent experiments. *p < 0.05, **p < 0.01.

To investigate the effect of circUBE2G1 on LUAD LN metastasis *in vivo*, a popliteal LN metastasis model was constructed by implanting mCherry-labeled circUBE2G1-overexpressing A549 cells into the footpads of nude mice (16). Immunohistochemistry (IHC) analysis of excised popliteal LNs demonstrated a higher metastatic rate in the circUBE2G1 overexpression group than in the control group (**Figures 4A–E**). Notably, circUBE2G1 overexpression significantly increased the density of LYVE-1-positive microlymphatic vessels (MLD) in the intratumoral and peritumoral areas of footpad primary tumor tissues, confirming that circUBE2G1 promotes lymphangiogenesis in LUAD (**Figures 4F, G**). In summary, these findings indicate that circUBE2G1 promotes lymphangiogenesis and LN metastasis of LUAD *in vivo*.

### 2.5 circUBE2G1 binds directly to hnRNPU in LUAD cells

**Figure 5:**
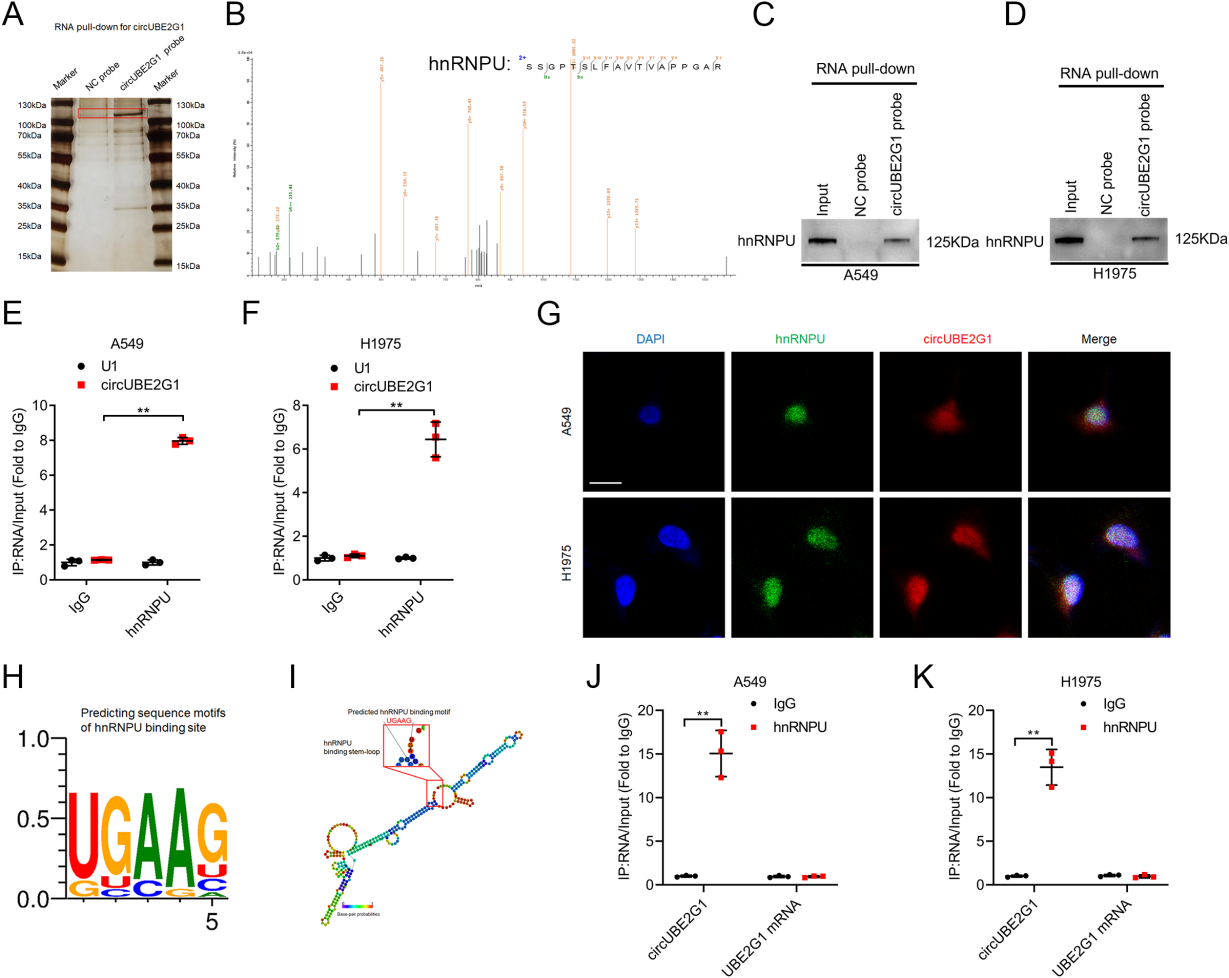
CircUBE2G1 directly binds to hnRNPU in LUAD cells. **(A)** Silver staining after pull-down with circUBE2G1 and control probes. **(B)** Mass spectrometry analysis of proteins binding to circUBE2G1 after RNA pull-down assay. **(C, D)** Western blotting showing the interaction between circUBE2G1 and hnRNPU in A549 **(C)** and H1975 **(D)** cells. **(E, F)** RIP assay revealing hnRNPU enrichment of circUBE2G1 in A549 **(E)** and H1975 **(F)** cells. **(G)** Representative images for the co-localization of circUBE2G1 and hnRNPU in LUAD cells. Scale bars, 5 μm. **(H)** RBPmap showing predicted hnRNPU-binding motifs. **(I)** Stem-loop structure of hnRNPU-binding motifs in circUBE2G1. **(J, K)** RIP assay after deleting circular binding sites of circUBE2G1 in A549 **(J)** and H1975 **(K)** cells. Statistical differences were assessed using two-tailed t-test in **(E, F, J, K)**. Error bars represent the standard deviation of three independent experiments. **p < 0.01.

Given the crucial role of circRNA cellular localization in its biological functions, the subcellular localization of circUBE2G1 was determined in LUAD cells (7). FISH and subcellular fraction assays revealed that circUBE2G1 predominantly existed in the cell nucleus (**Supplementary Figures S1F–H**). Nuclear circRNAs mainly exert their biological functions by interacting with RNA-binding proteins (17, 18). Thus, a biotinylated probe targeting circUBE2G1 was employed for RNA pull-down assay and to identify the interacting proteins. Compared to the control group, silver staining revealed distinct bands in the biotinylated circUBE2G1 group, with a molecular weight of 100-130 kDa (**Figure 5A**). Mass spectrometry (MS) and Western blotting revealed that the band was hnRNPU (**Figures 5B–D**). Consistently, RNA immunoprecipitation (RIP) assays using an anti-hnRNPU antibody demonstrated significant enrichment of circUBE2G1 compared to the IgG control group (**Figures 5E, F**; **Supplementary Figures S1I–K**). Confocal fluorescence microscopy indicated the co-localization of circUBE2G1 and hnRNPU in the nucleus of LUAD cells (**Figure 5G**). To pinpoint the specific interaction sites between circUBE2G1 and hnRNPU, the RBPmap website (a website for mapping the binding sites of RNA-binding proteins) was utilized to identify the loop-forming regions of circUBE2G1 (**Figures 5H, I**). Linear UBE2G1 mRNA significantly impaired the enrichment of hnRNPU (**Figures 5J, K**), indicating the crucial role of the circular binding site sequence in circUBE2G1-hnRNPU interaction.

### 2.6 circUBE2G1 promotes VEGF-C transcription by recruiting hnRNPU

**Figure 6:**
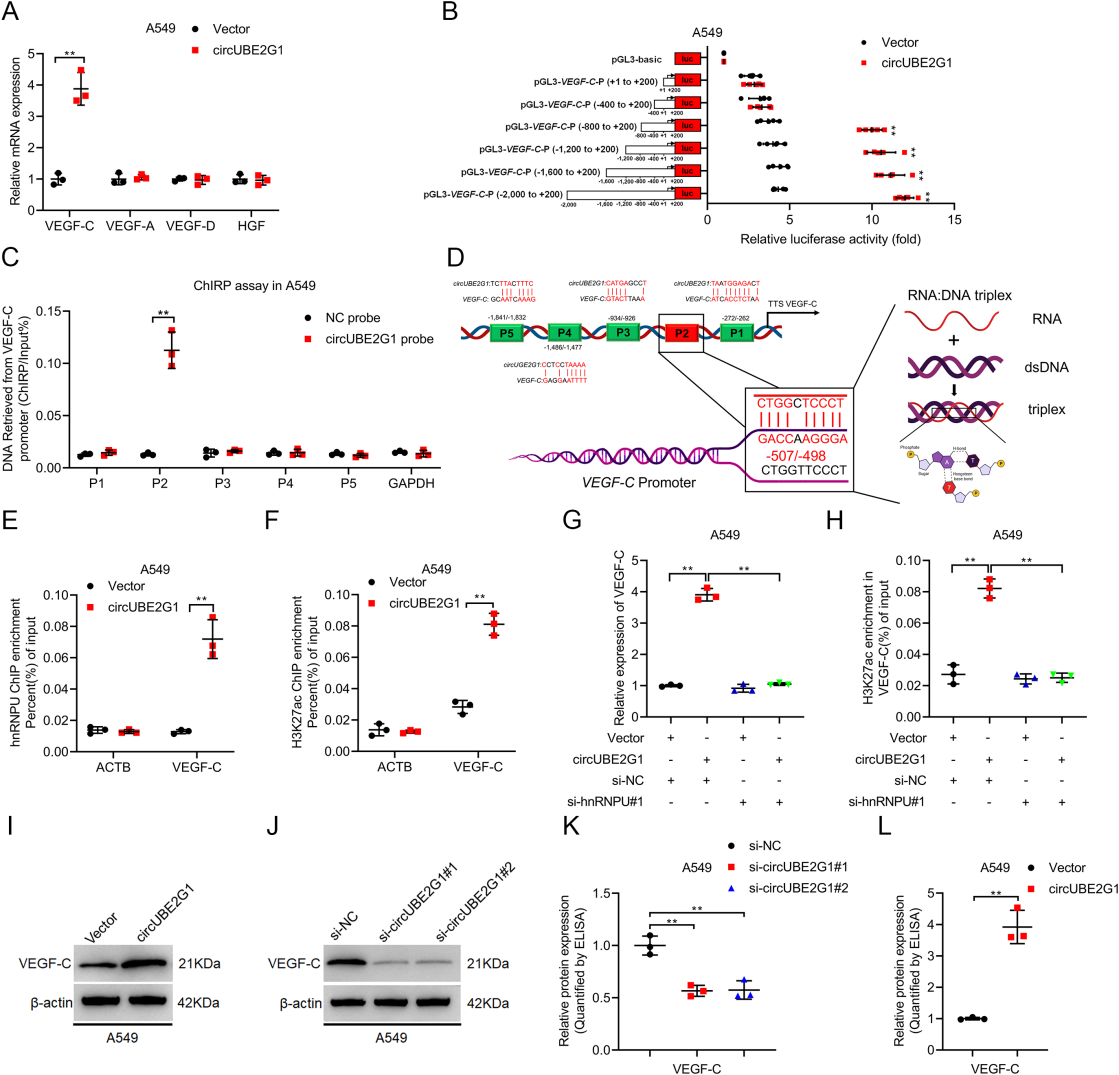
CircUBE2G1 recruits hnRNPU to promote VEGF-C transcription. **(A)** qRT-PCR analysis measuring changes in lymphangiogenic factors after circUBE2G1 overexpression in A549 cells. **(B)** Transcriptional activity of VEGF-C in circUBE2G1-overexpressing A549 cells transfected with truncated VEGF-C promoter luciferase plasmids. **(C)** ChIRP assay detecting circUBE2G1-associated chromatin fragments of the VEGF-C promoter in A549 cells. **(D)** Schematic illustration of the DNA-RNA triplex structure between circUBE2G1 and the VEGF-C promoter. **(E, F)** ChIP-qPCR showing hnRNPU **(E)** and H3K27ac **(F)** enrichment on the VEGF-C promoter after circUBE2G1 overexpression in A549 cells. **(G)** qRT-PCR analysis of VEGF-C expression in circUBE2G1-overexpressing A549 cells with or without hnRNPU silencing. **(H)** ChIP-qPCR of H3K27ac enrichment on the VEGF-C promoter in circUBE2G1-overexpressing A549 cells with or without hnRNPU silencing. **(I, J)** Western blotting for VEGF-C expression. **(K, L)** ELISA for measuring the effect of circUBE2G1 on VEGF-C secretion. Statistical significance was assessed using one-way ANOVA followed by Dunnett’s tests in **(A)**. Statistical difference was assessed using two-tailed student t-test in **(A–C, E, F, K, L)**, and one-way ANOVA followed by Dunnett tests in **(G, H)**. Error bars show the standard deviation from three independent experiments. **p < 0.01.

The expression profile of key lymphangiogenic factors was investigated to identify the target genes of circUBE2G1. VEGF-C was significantly upregulated in LUAD cells overexpressing circUBE2G1 and downregulated after circUBE2G1 knockdown (**Figure 6A**; **Supplementary Figures S2A–C**). Luciferase reporter plasmids containing varying lengths (i.e., -2000 nt to +200 nt) of the VEGF-C promoter sequence were constructed to delve deeper into the regulatory mechanism by which circUBE2G1 upregulates VEGF-C expression. circUBE2G1 overexpression markedly increased the transcriptional activity of the -400 to -800 nt sequence in the VEGF-C promoter (**Figure 6B**; **Supplementary Figure S2D**). Chromatin isolation using the RNA purification (ChIRP) assay confirmed the interaction between circUBE2G1 and the P2 region of the VEGF-C promoter (**Figure 6C**; **Supplementary Figure S2E**). Sequence comparative analysis predicted a complementary region (-498 to -507 nt) between circUBE2G1 and the VEGF-C promoter (**Figure 6D**). In fact, mutation of this region strongly reduced the ability of circUBE2G1 to upregulate the luciferase activity of the VEGF-C promoter (**Supplementary Figures S2F, G**).

Histone modifications play a pivotal role in epigenetic modification. It has been reported that hnRNPU activates gene transcription by acetylating H3K27 (H3K27ac) (19, 20). Therefore, we investigated whether circUBE2G1 promotes VEGF-C transcription by recruiting hnRNPU to induce H3K27ac on the VEGF-C promoter. Chromatin immunoprecipitation (ChIP) assay showed that in LUAD cells overexpressing circUBE2G1, the enrichment of hnRNPU and H3K27ac significantly increased on the VEGF-C promoter (**Figures 6E, F**; **Supplementary Figures S2H, I**). Furthermore, ChIP analysis demonstrated that silencing hnRNPU significantly attenuated circUBE2G1 overexpression-induced VEGF-C transcription and H3K27ac enrichment on the VEGF-C promoter (**Figures 6G, H**; **Supplementary Figures S2J, K**). VEGF-C was significantly upregulated after circUBE2G1 overexpression (**Figures 6I–L**; **Supplementary Figures S2L–O**), while the expression of VEGF-C was downregulated after circUBE2G1 knockdown in LUAD cells. In conclusion, our results suggest that circUBE2G1 activates VEGF-C transcription by recruiting hnRNPU to enhance H3K27ac on the VEGF-C promoter.

### 2.7 circUBE2G1 enhances lymphangiogenesis by stimulating VEGF-C secretion in LUAD

**Figure 7:**
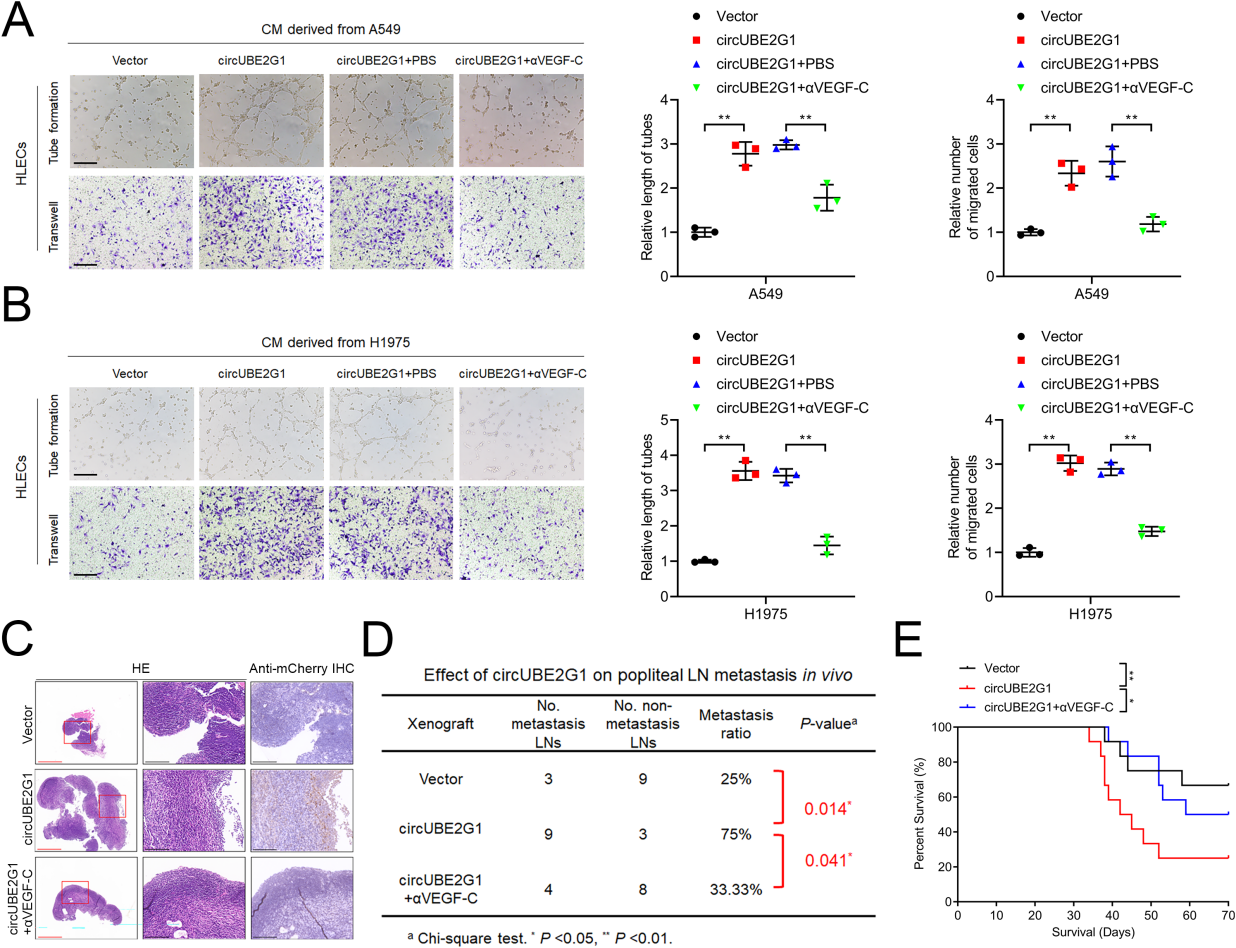
CircUBE2G1 promotes lymphangiogenesis and LN metastasis in LUAD by enhancing VEGF-C expression. **(A, B)** Representative images and quantification of tube formation and migration of HLECs induced by conditioned medium from A549 **(A)** or H1975 **(B)** cells under different treatments. Scale bars, 100 μm. **(C)** Representative images of anti-mCherry immunohistochemical staining of popliteal LNs in nude mice (*n* = 12 per group). Red scale bars, 500 μm; black scale bars, 100 μm. **(D)** Popliteal LN metastasis rate across all groups (*n* = 12 per group). **(E)** Kaplan-Meier survival curves (*n* = 12 per group). Statistical differences were evaluated using the two-tailed student t-test in **(A, B)** and the χ2 test in **(D)**. Error bars represent the standard deviation for three independent experiments. *P < 0.05; **P < 0.01.

Next, we assessed whether circUBE2G1-mediated VEGF-C expression promotes lymphangiogenesis and LN metastasis in LUAD. VEGF-C blockade with the neutralizing antibody pV1006R-r significantly inhibited the tube formation and migration of HLECs induced by circUBE2G1 overexpression (**Figures 7A, B**). Consistently, *in vivo* experiments revealed that circUBE2G1 overexpression enhanced lymph node metastasis, while treatment with pV1006R-r inhibited LN metastasis mediated by circUBE2G1 overexpression (**Figure 7C**). Furthermore, pV1006R-r reduced the LN metastasis rate in mice with circUBE2G1-overexpressing tumor, significantly extending the survival time (**Figures 7D, E**). Overall, these results demonstrate that circUBE2G1 promotes LN metastasis in LUAD by recruiting hnRNPU and promoting VEGF-C expression.

## 3 Discussion

LN metastasis is a critical determinant for staging patients with lung cancer and selecting optimal treatment strategies, serving as one of the most reliable prognostic indicators for these patients (21, 22). Once lymphatic metastasis occurs, the five-year survival rate of patients drops from 75% to 20%. Previous studies have shown that lymphangiogenesis is closely associated with tumor lymphatic metastasis through the expansion of the lymphatic drainage (16). Nevertheless, the molecular mechanisms underlying lymphangiogenesis in LUAD remain elusive. This study identified a circRNA, circUBE2G1, which is upregulated in LUAD and positively correlates with lymphangiogenesis and LN metastasis. We confirmed that knocking out circUBE2G1 could inhibit lymphatic metastasis in LUAD via *in vivo* and *in vitro* study. Mechanistically, circUBE2G1 recruits hnRNPU to enhance H3K27ac on the VEGF-C promoter, thereby activating VEGF-C transcription and promoting lymphangiogenesis and LN metastasis in LUAD. Our findings underscore the role of circRNAs in regulating lymphangiogenesis and lymphatic metastasis in LUAD and suggest that targeting circUBE2G1 may represent a potential therapeutic strategy for LN metastasis in LUAD.

Previous studies have identified VEGF-C as a critical stimulator of lymphangiogenesis, playing a pivotal role in tumor growth, invasion, and metastasis (23, 24). The binding of VEGF-C to VEGFR-3 activates downstream signaling pathways, promoting lymphatic endothelial cell migration, proliferation, and survival (25, 26). Previous studies mainly focused on the regulation of VEGF-C secretion, however, the transcriptional regulatory mechanisms involved in VEGF-C remain unclear.In this study, we identified at least one regulatory pathway. HnRNPU is recruited by circUBE2G1 and then directly interacts with the VEGF-C promoter, inducing H3K27ac to activate VEGF-C transcription. Mutations in the hnRNPU-binding site within VEGF-C significantly inhibited its activation, impairing lymphatic metastasis in LUAD. We uncovered a novel mechanism in which the transcription regulation of VEGF-C was mediated by circRNA. Our findings provide evidence for targeting VEGF-C in LUAD with LN metastasis.

Initially considered functionless, circRNAs have recently gained attention due to their importance in tumor progression (27). In previous studies, circRNAs mainly act as sponges for microRNAs in tumorigenesis (28). For instance, in renal cell carcinoma, circMYLK functions as a competing endogenous RNA (ceRNA) against miR-513a-5p predominantly in the cytoplasm, thereby inducing VEGF-C expression and promoting metastasis (29). Recently, increasing evidences have confirmed that circRNAs exert their functions through protein regulation (30). In Yao’s work, circ_0026611 enhances lymphangiogenesis in esophageal squamous cell carcinoma (ESCC) by inhibiting the acetylation and ubiquitination of PROX1 (31). However, the binding partners and regulatory mechanisms of circRNAs in LN metastasis of LUAD remain unclear. In this study, we demonstrated that circUBE2G1 interacts with hnRNPU and regulates the transcription of its target gene VEGF-C by recruiting hnRNPU, thus promoting LN metastasis of LUAD. It has been reported that circUBE2G1 is widely expressed in various human cancers, including gastric cancer, lung squamous cell carcinoma, and hepatocellular carcinoma (32, 33). Our results confirmed that the interaction between circUBE2G1 and hnRNPU is also present in these tumors. This indicates that circUBE2G1 may exert oncogenic effects in a wider range of human cancers by binding with hnRNPU, highlighting its potential as a promising therapeutic target.

In summary, this study identified a novel mechanism by which a circRNA upregulates VEGF-C to promote LN metastasis in LUAD. Our clinical findings indicated that upregulation of circUBE2G1 is associated with poor prognosis in patients with LUAD. Furthermore, it was suggested that circUBE2G1 overexpression can promote VEGF-C transcription by recruiting hnRNPU, thereby facilitating LN metastasis in LUAD. Identification of circUBE2G1 expands our understanding of LN metastasis and provides a potential therapeutic target for LN metastasis in LUAD.

## 4 Materials and methods

### 4.1 Clinical samples and ethics statement

Tumor tissues and paired adjacent tissues were collected from 154 patients with LUAD who underwent surgery at Sun Yat-sen Memorial Hospital of Sun Yat-sen University (Guangzhou, Guangdong, China). The histopathological type of each clinical sample was independently diagnosed by three pathologists. Clinical data were collected after obtaining informed consent from patients. The Ethics Committee of Sun Yat-sen Memorial Hospital of Sun Yat-sen University approved this study (Ethics No.: SYSKY-2023-1040-01). This study strictly followed the available ethical guidelines.

### 4.2 Cell lines and cell culture

Human LUAD cell lines A549, H520 and H1975 were purchased from the American Type Culture Collection (ATCC, Manassas, VA, USA) and cultured in Dulbecco’s Modified Eagle Medium, DMEM (Gibco, cat# C11995500BT) and Roswell Park Memorial Institute (RPMI) 1640 medium (Gibco, cat# C11875500BT), respectively, supplemented with 10% fetal bovine serum (FBS) (BI, cat# 04–001–1ACS). Gastric adenocarcinoma cell lines AGS were purchased from the ATCC and cultured in RPMI 1640 medium with 10% FBS. Human hepatocellular carcinomas (HepG2) cells lines were purchased from the ATCC and cultured in DMEM medium with 10% FBS. Human lymphatic endothelial cells (HLECs) were purchased from ScienCell Research Laboratories (Carlsbad, California, USA, Cat# 2500) and cultured in endothelial cell medium (ECM; ScienCell Research Laboratories, Cat# 1001) supplemented with 5% FBS. All cell lines were authenticated by short tandem repeat (STR) profiling and were negative for mycoplasma contamination.

### 4.3 Mouse model of popliteal lymphatic metastasis

mCherry-labeled A549 cells (5×10^5^) suspended in 20 μl of phosphate-buffered saline (PBS) were injected into the footpads of 4-5-week-old BALB/c nude mice (Beijing Vital River Laboratories Animal Technology, Beijing, China). Mice were euthanized when the tumor volume reached 200 mm^3^, and footpad tumors and popliteal lymph nodes were fixed in formalin for subsequent experiments. Animal experiments were approved by the Institutional Animal Care and Use Committee and complied with relevant national regulations for the welfare of experimental animals.

### 4.4 RNA pull-down assay

Cells were lysed using the Magnetic RNA-Protein Pull-Down Kit (Thermo Scientific, catalog no. 20164), immediately frozen in liquid nitrogen, and stored at -80°C for at least 2 hours. Streptavidin-labeled magnetic beads (Invitrogen, catalog no. 88817) were incubated with biotinylated probes against circUBE2G1 (Genepharma), washed, and incubated overnight at 4°C, with the cell lysate supernatant collected by centrifugation. Samples were collected for subsequent analyses after washing and elution of proteins.

### 4.5 *In situ* hybridization

ISH was performed using the Enhanced Sensitive ISH Detection Kit II (Boster Biological Technology, Pleasanton, CA, USA, Cat# MK1032) following the manufacturer’s instructions. Paraffin sections were deparaffinized and digested using proteinase K. Tissues were pretreated with RNase A for 4 hours at 37°C and then hybridization with probe at 37°C overnight. Staining was done and imaging was performed using a Nikon Eclipse 80i microscope (Nikon, Tokyo, Japan). **Supplementary Table S1** shows the circUBE2G1 probe sequences used for the ISH.

### 4.6 RNA immunoprecipitation

RIP was performed using the EZ-Magna RIP kit (Merck, Darmstadt, Germany, Cat#17-701) following the manufacturer’s instructions. Briefly, 2x10^7^ LUAD cells were lysed, and the supernatant was collected after centrifugation and incubated overnight with magnetic beads conjugated with anti-hnRNPU antibody. RNA was eluted and collected for analysis after one day. **Supplementary Table S2** lists the detailed antibodies used in the experiments.

### 4.7 Chromatin immunoprecipitation

ChIP was performed using the EZ-Magna ChIP A/G kit (Millipore, Billerica, MA, USA, Cat#17-371) following the manufacturer’s instructions. Briefly, 1x10^7^ LUAD cells were collected after centrifugation. Chromatin was isolated after cross-linking with 4% paraformaldehyde and lysing with lysis buffer. Subsequently, chromatin was sonicated to shear chromatin into fragments of 500–800 bp in length. These fragments were incubated with hnRNPU or H3K27ac antibodies. Antibody-chromatin complexes were immunoprecipitated overnight with protein A/G-coated magnetic beads. DNA was eluted and collected for analysis on the following day. Normal rabbit IgG was used as the negative control. **Supplementary Table S1** lists the detailed antibodies used in the experiments.

### 4.8 Chromatin isolation by RNA purification

ChIRP was performed using the Magna ChIRP RNA Interactome Kit (Millipore, Cat#17-10494) following the manufacturer’s instructions. Chromatin fragments (200–1000 bp) from 4x10^7^ LUAD cells were obtained after fixation, lysis, and sonication. They were incubated with biotinylated probes against circUBE2G1. The probe-chromatin complexes were precipitated overnight with streptavidin-conjugated magnetic beads at 4°C. After washing, the combined DNA was collected for analysis. **Supplementary Table S1** lists the detailed sequences of the probes.

### 4.9 Tube formation assays

Matrigel (BD Biosciences, Franklin Lakes, NJ, USA, Cat#356234) mixed with serum-free ECM medium (1:2) was seeded in a 24-well plate (300 μl per well). After gelation, 1x10^5^ HLECs were seeded per well and incubated at 37°C for 4 hours. Lymphatic tube images were captured using an inverted fluorescence microscope, and the tube length was analyzed using ImageJ software (NIH, Bethesda, MD, USA, RRID: SCR_003070).

### 4.10 Statistical analysis

Data were analyzed using GraphPad Prism 9 (GraphPad Software, La Jolla, CA, USA) and SPSS 26.0 (IBM, Chicago, IL, USA). Quantitative data are presented as mean ± standard deviation of at least three independent experiments. Differences were assessed using t-test or one-way analysis of variance. In addition, Dunnett’s test was employed for continuous variables, chi-square test was employed for categorical variables, Pearson correlation analysis was employed to assess correlations, and Kaplan–Meier survival analysis and log-rank test were employed to measure overall survival (OS) and disease-free survival (DFS).

## Data Availability

The original contributions presented in the study are included in the article/**Supplementary Material**. Further inquiries can be directed to the corresponding authors.
