# Supplementary material for: circUBE2G1 interacts with hnRNPU to promote VEGF-C-mediated lymph node metastasis of lung adenocarcinoma
Source: Front Oncol. 2024 Nov 27;14:1455909. doi: 10.3389/fonc.2024.1455909 (PMC11631705; doi:10.3389/fonc.2024.1455909)
Supplement: Supplementary file 1 [file DataSheet1.docx]

Supplementary Material

# Supplementary Methods

## Immunohistochemistry (IHC)

Paraffin-embedded tissue sections were deparaffinized and rehydrated in a series of xylene and graded alcohol concentrations after baking at 60°C for 2 hours. Antigen retrieval was performed using ethylenediaminetetraacetic acid (EDTA) buffer. Endogenous peroxidase activity was blocked with 3% hydrogen peroxide, and non-specific antigen sites were blocked with sheep serum. Primary antibodies were incubated overnight at 4°C. On the following day, after incubation with secondary antibodies at room temperature for 30 minutes, sections were stained with 3, 3'-diaminobenzidine (DAB) and hematoxylin staining solutions. Dehydration was conducted using graded alcohol and xylene, and neutral gum mounting mediums were air-dried and observed using a Nikon Eclipse 80i microscope (Nikon, Tokyo, Japan).

## Luciferase reporter assays with serial fragments of the vascular endothelial growth factor C (VEGF-C) promoter

Truncated sequences from +200 to -2000 bp of the VEGF-C promoter were cloned into the pGL3-basic luciferase reporter vector (Igebio, Guangzhou, China). The luciferase reporter plasmid was co-transfected into LUAD cells. After 48 hours of incubation, luciferase activity was measured using the dual-luciferase reporter assay system (Promega, Madison, WI, USA, Cat#E1910), normalized to the ratio of firefly and Renilla luciferase activity.

## Proteins extraction and Western blotting

Total proteins were isolated at 48 hours after LUAD cells transfection using radioimmunoprecipitation assay (RIPA) lysis buffer supplemented with protease and phosphatase inhibitors. Protein concentrations were determined using the bicinchoninic acid (BCA) protein assay kit. Western blotting was performed to analyze the expression of target proteins. Briefly, 20 μg of total protein was separated using 10% sodium dodecyl sulfate-polyacrylamide gel electrophoresis (SDS-PAGE) gel electrophoresis and transferred to polyvinylidene fluoride (PVDF) membranes. After blocking with 5% bovine serum albumin (BSA), membranes were incubated overnight with primary antibodies at 4°C, followed by incubation with horseradish peroxidase (HRP)-conjugated secondary antibodies at room temperature for 1 hour. Protein bands were visualized using a super-sensitive electrochemiluminescence (ECL) solution (Thermo Scientific, Cat# 32209), and densitometric analysis was performed using Image J software.

## RNA extraction and qRT-PCR

Total RNA was extracted using Trizol reagent (Takara Bio, Japan, Cat#9109). cDNA was synthesized using the Hiscript III Reverse Transcriptase kit (Vazyme, Nanjing, China, Cat#R312-01), and qRT-PCR was performed using the ChamQTM Universal SYBR qPCR Master Mix kit (Vazyme, Cat#Q711-02) to evaluate the mRNA levels of target genes.

## RNase R treatment and actinomycin D assays

Total RNA (2 μg) was treated with or without 6 U RNase R (Geneseed Biotech, Guangzhou, China, catalog no. R0301) for 30 minutes at 37°C, followed by qRT-PCR.

80% confluent LUAD cells were treated with 5 μg/ml actinomycin D (APExBIO, Houston, TX, USA, Cat#A4448) in six-well plates, and total RNA was collected at specific time points (6, 12, 18, and 24 hours).

## Plasmids and siRNA transfection

Overexpression plasmids for CircUBE2G1 and hnRNPU were constructed using the pcDNA3.1 vector (RRID: Addgene_79663) by Igebio (Guangzhou, China). SiRNAs were purchased from Igebio to downregulate circUBE2G1 and hnRNPU. The day before transfection, seed 2 x 10^5^ cells in 2.5 mL growth medium for a single well of a 6-well plate. The cell culture must be 80% confluent and have >90% viability on the day of transfection. Transfection was performed using the Lipofectamine 3000 kit (Invitrogen, Cat# L3000015) following the manufacturer's instructions. Transfection efficiency was validated using qRT-PCR and Western blotting.

## Subcellular fraction assays

Subcellular fractions were isolated using the PARISTM kit (Thermo Scientific, Cat#AM1921) following the manufacturer's instructions. Briefly, 1 x 10^6^ LUAD cells were centrifuged to collect the cell pellet. The cell pellet was then lysed using cell fractionation buffer and cell disruption buffer to obtain separated cytoplasmic and nuclear fractions. RNA was extracted using Trizol reagents (Takara Bio, Cat#9109) and analyzed by qRT-PCR. 18S rRNA, serving as a nuclear reference, and U1, serving as a cytoplasmic reference, were employed for quality control by qRT-PCR analysis. Abundant 18S rRNA and rare U1 detected in cytoplasmic fraction while high enrichment U1 and rare 18S rRNA in the nuclear fraction indicates the separation of high purity of nuclear or cytoplasmic fractions.

## Transwell assays

The lower chamber of Transwell chambers (Corning Costar Corp, USA, Cat#3422) contained 700 μl of ECM medium supplemented with serum, and the upper chamber was seeded with 1x10^5^ HLECs suspended in 300 μl of serum-free ECM medium. After 4-6 hours of incubation, cells were fixed with 4% paraformaldehyde for 10 minutes and stained with crystal violet for 20 minutes. Images were captured using a Nikon Eclipse 80i microscope (Nikon).

## RNA fluorescence in situ hybridization (FISH)

Cells seeded in confocal dishes were fixed with 4% paraformaldehyde for 10 minutes and permeabilized with 0.5% Triton X-100 for 10 minutes. Cy3-labeled probes against circUBE2G1 (GenePharma, Suzhou, China) were hybridized with cells overnight using the fluorescence in situ hybridization (FISH) Kit (RiboBio, Guangzhou, China, Cat#C10910). 4′,6-Diamidino-2-phenylindole (DAPI) was used for nuclear staining, and images were captured using a Zeiss Lam 710 confocal microscope (Carl ZEISS AG, Oberkochen, Germany).

## Immunofluorescence (IF) assays

Cells cultured in confocal dishes were fixed with 4% paraformaldehyde for 10 minutes after reaching approximately 80% confluence. After washing three times with pre-cooled PBS, cells were permeabilized with 0.5% Triton X-100 for 15 minutes at room temperature. After 1 hour of blocking with 5% BSA at room temperature, primary antibodies were incubated at 4°C overnight. The next day, after 1 hour of incubation with fluorescent secondary antibodies at room temperature and 15 minutes of DAPI staining, images were captured using a ZEISS Lam 710 focal microscope (Carl Zeiss AG).

## Silver staining

Following RNA pull-down, equal volumes of protein samples were separated using 10% SDS-PAGE gel electrophoresis. Then, the samples were subjected to silver staining using the Silver Staining Kit (Thermo Scientific, Cat#24612) following the manufacturer's instructions. Briefly，the gel was washed in ultrapure water and then fixed using fixation solution containing 30% ethanol and 10% acetic acid for 30 minutes. After fixation, the gel was washed in 10% ethanol solution and ultrapure water separately. Sensitizer Working Solution (1:500 dilution) was added to the gel and incubated for exactly 1 minute, then washed with two changes of ultrapure water for 1 minute each. Stain Working Solution (1:50 dilution) was added and incubated for 30 minutes followed by adding Developer Working Solution (1:50 dilution) and incubated until protein bands appear (2-3 minutes). When the desired band intensity was reached, Stop Solution (5% acetic acid) was added to stop the reaction.

## ELISA

Enzyme-linked immunosorbent assay (ELISA) was conducted following the instructions of the Human VEGF-C ELISA Kit (Proteintech, Cat. No.: KE00252). 72 hours after transfection, the cultured media of LUAD cells were collected and 100 μl of them were added into the microwells followed with the incubation of VEGF-C antibody at 37℃ for 2h. VEGF-C protein is detected by VEGF-C specific biotinylated antibody after extensive washing. Tetramethyl-benzidine (TMB) reagent is used to develop the signal. Solution containing sulfuric acid is used to stop color development and the color intensity is measurable at 450 nm with the correction wavelength set at 630 nm. Absorbance was read using a PerkinElmer VICTOR™ X3 Multilabel Plate Reader.

# Supplementary Figures and Tables

## Supplementary Figures


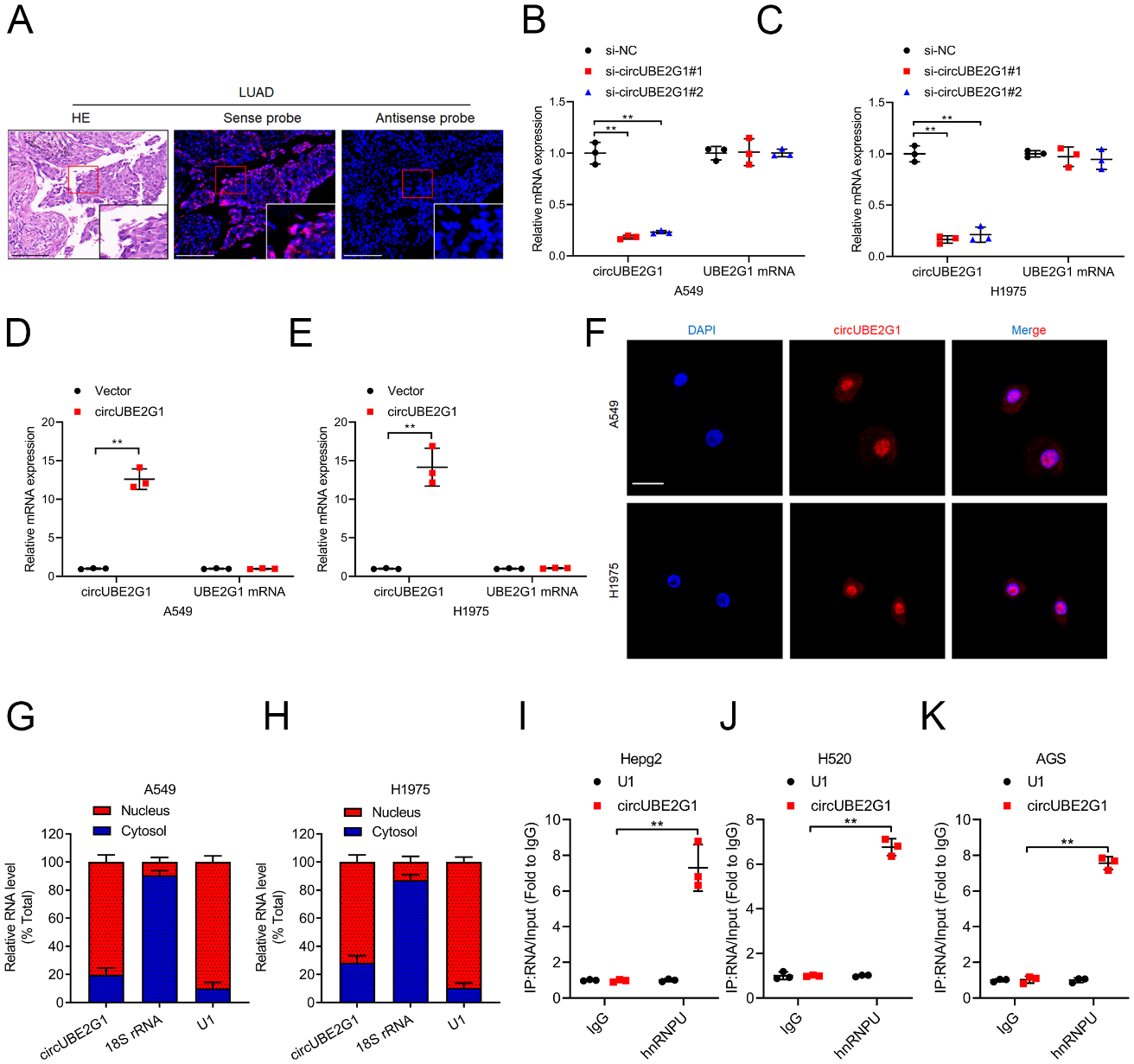


**Supplementary Figure 1. (A)** Representative FISH images of the antisense probe as negative control and sense probe as positive control. **(B-E)** Transfection efficiency of circUBE2G1 in LUAD cell lines. **(F-H)** FISH **(F)** and subcellular fraction analysis **(G-H)** of circUBE2G1 in LUAD cells. **(I-K)** RIP assay revealing hnRNPU enrichment of circUBE2G1 in Hepg2 (**I**), H520 (**J**) and AGS (**K**) cells. Scale bars, 5 μm. Statistical differences were assessed using two-tailed student t-test **(B-E and I-K)**. Error bars represent the standard deviation from three independent experiments. **P < 0.05; **P < 0.01.*


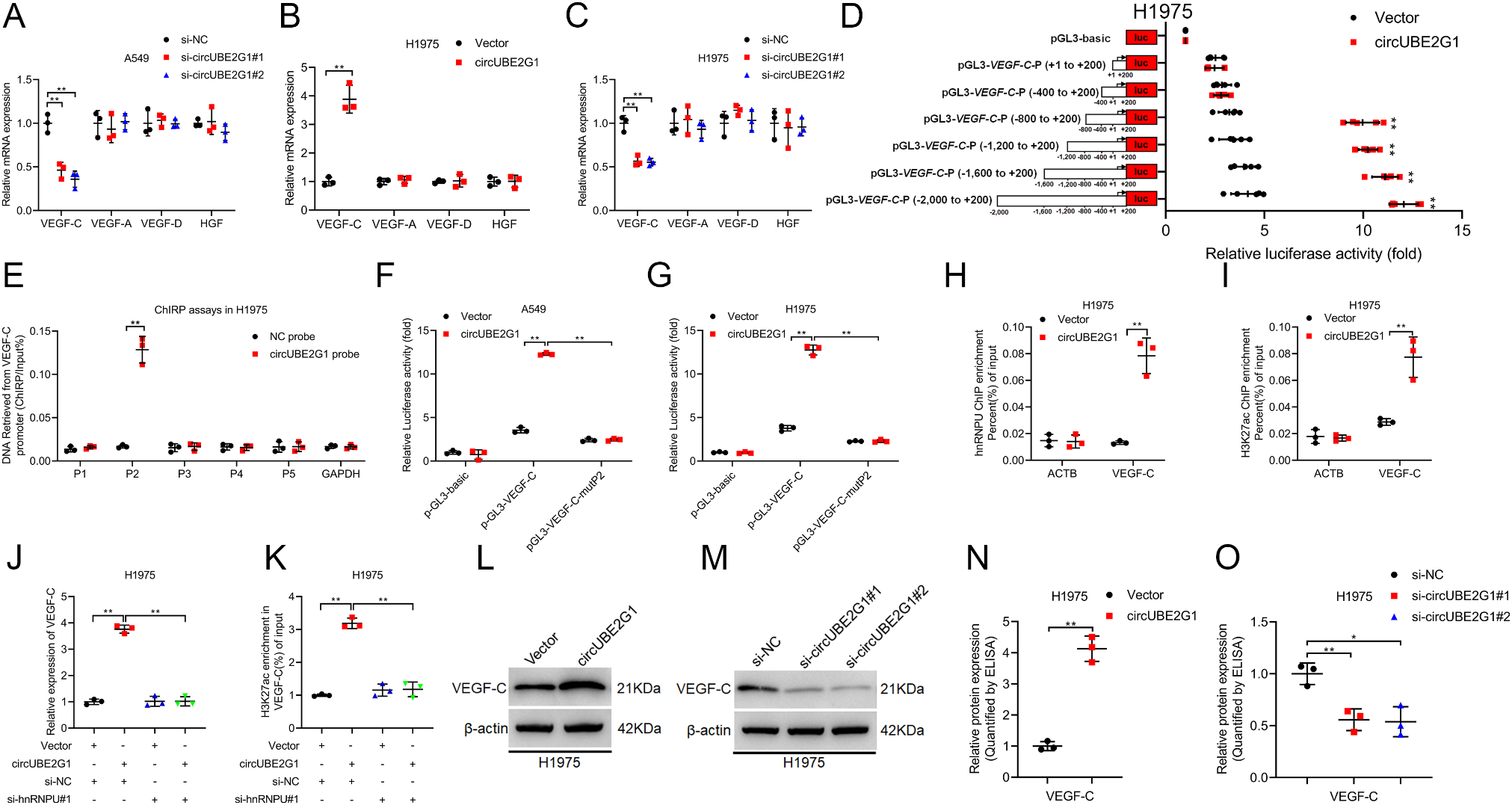


**Supplementary Figure 2.** CircUBE2G1 activates VEGF-C transcription by increasing H3K27ac on the VEGF-C promoter. **(A-C)** qRT-PCR analysis measuring changes in the expression of lymphangiogenic factors in LUAD cells with circUBE2G1 overexpression or silencing. **(D)** VEGF-C transcription in H1975 cells overexpressing circUBE2G1 transfected with truncated VEGF-C promoter luciferase plasmids. **(E)** ChIRP assay detecting circUBE2G1-associated chromatin fragments of the VEGF-C promoter in A549 cells. **(F-G)** Luciferase activity in A549 **(F)** and H1975 **(G)** cells. **(H-I)** ChIP-qPCR showing hnRNPU **(H)** and H3K27ac **(I)** enrichment on the VEGF-C promoter after circUBE2G1 overexpression in H1975 cells. **(J)** qRT-PCR analysis of VEGF-C expression in H1975 cells overexpressing circUBE2G1 with or without hnRNPU silencing. **(K)** ChIP-qPCR of H3K27ac enrichment on the VEGF-C promoter in H1975 cells overexpressing circUBE2G1 with or without hnRNPU silencing. **(L-M)** Western blotting of VEGF-C. **(N-O)** ELISA measuring the effect of circUBE2G1 on VEGF-C secretion. The statistical difference was assessed using two-tailed student t-test in **(A-E, H, I, N, and O)**, and one-way ANOVA followed by Dunnett tests in **(F, G, J, and K)**. Error bars represent the standard deviation from three independent experiments. **P < 0.05; **P < 0.01.*

## Supplementary Tables

**Supplementary Table S1.** Primers and probes used in this study.

| **Target gene** | **Sequence (5’-3’)** | **Application** |
| --- | --- | --- |
| CircUBE2G1 | F: ATGAAAAGCCAGAGGAACGC  R: AGGAGGTCGGAGGGGATAAT | qRT-PCR |
| UBE2G1 | F: AGGTGGTGTTTTTAAGGCTCATC  R: CATTTGGGTGCCAGATTTCTGTA | qRT-PCR |
| U1 | F: CAGGGGAGATAACGTGACCA  R: GGGAAAAGCACGGACACAG | qRT-PCR  RIP |
| 18S rRNA | F: AGCAGACATTGACCTCACCA  R: CCTCTATGGGCCCGAATCTT | qRT-PCR |
| GAPDH | F: CAAATTCCATGGCACCGTCA  R: ATGATGTTCTGGAGAGCCCC | qRT-PCR  ChIRP |
| ACTB | F: TGACAAAACCTAACTTGCGCA  R: CAATCAAAGTCCTCGGCCAC | ChIP |
| VEGF-C | F: TGGGGAAGGAGTTTGGAGTC  R: GTTACTGGTTTGGGGCCTTG | qRT-PCR |
| VEGF-A | F：CAGGCTGCTGTAACGATGAA  R：GCATTCACATCTGCTGTGCT | qRT-PCR |
| VEGF-D | F: ACCTTCCATTCACACCAGCT  R: GACACCTGCCATTCCATGAC | qRT-PCR |
| HGF | F: CAAGCAATCCAGAGGTACGC  R: AGTATAGCACCATGGCCTCG | qRT-PCR |
| Si-circUBE2G1#1 | Sense: AUGCUGCGUGAAGGUGGUGUU  Antisense: AACACCACCUUCACGCAGCAU | Si-RNA |
| Si-circUBE2G1#2 | Sense: UGCUGCGUGAAGGUGGUGUUU  Antisense: AAACACCACCUUCACGCAGCA | Si-RNA |
| CircUBE2G1 probe | 5'(Biotin)AAAACACCACCTTCACgCAgCATCAACATTA(Biotin)-3' | Pull down |
|  | 5’-Cy3 labeled and 3’-Cy3 labeled | FISH |

**Supplementary Table S2.** Antibodies used in this study.

| **Product** | **Source** | **Catalog no.** | **Dilution** |
| --- | --- | --- | --- |
| **Primary antibody** |  |  |  |
| ***Western blotting*** |  |  |  |
| anti-hnRNPU | Cell Signaling Technology | 34095 | 1:1000 |
| Anti-VEGF-C | Cell Signaling Technology | 2445 | 1:1000 |
| anti-H3K27ac | Abcam | Ab4729 | 1:1000 |
| anti-β-actin | Abcam | ab8226 | 1:5000 |
| ***Immunohistochemistry*** |  |  |  |
| anti-LYVE-1 | Abcam | ab218535 | 1:500 |
| anti-mCherry | Abcam | ab125096 | 1:2000 |
| ***Immunofluorescence*** |  |  |  |
| anti-hnRNPU | Cell Signaling Technology | 34095 | 1:200 |
| anti-LYVE-1 | Abcam | ab218535 | 1:500 |
| ***Immunoprecipitation*** |  |  |  |
| anti-hnRNPU | Cell Signaling Technology | 34095 | 10 µg for 10^7^ cells lysate |
| anti-H3K27ac | Abcam | Ab4729 | 2 µg for 25 µg of chromatin. |
| **Secondary antibody:** |  |  |  |
| ***Western blotting*** |  |  |  |
| anti-rabbit IgG-HRP | Cell Signaling Technology | 7074 | 1:5000 |
| anti-mouse IgG-HRP | Cell Signaling Technology | 7076 | 1:5000 |
| ***Immunohistochemistry*** |  |  |  |
| anti-rabbit IgG-HRP | Proteintech | SA00001-2 | 1:10000 |
| anti-mouse IgG-HRP | Proteintech | SA00001-1 | 1:10000 |
| ***Immunofluorescence*** |  |  |  |
| [Alexa Fluor 4](https://www.baidu.com/link?url=nF9d2Xaur7vyZuSh6bwYgXJHxCoqgi5ljmVkB6q--I4j4E8mmfQwWu1WHii3mT9LmMQ5XQE23xsmXGJKAUgtmctb7vuX9L1odcCwYmhMNRgtKDKfaFBBlPGw7dU_i5LpQii0iVI7_AAuccqxBIw54_&wd=&eqid=cad6a7aa0006a5040000000661054afd)88 | Invitrogen | A32766 | 1:1000 |
| [Alexa Fluor 555](https://www.baidu.com/link?url=nF9d2Xaur7vyZuSh6bwYgXJHxCoqgi5ljmVkB6q--I4j4E8mmfQwWu1WHii3mT9LmMQ5XQE23xsmXGJKAUgtmctb7vuX9L1odcCwYmhMNRgtKDKfaFBBlPGw7dU_i5LpQii0iVI7_AAuccqxBIw54_&wd=&eqid=cad6a7aa0006a5040000000661054afd) | Invitrogen | A32773 | 1:1000 |
